# The trophoblast giant cells of cricetid rodents

**DOI:** 10.3389/fcell.2022.1097854

**Published:** 2023-01-16

**Authors:** Phelipe O. Favaron, Anthony M. Carter

**Affiliations:** ^**1**^ Department of General Biology, Biological Sciences Center, State University of Londrina, Paraná, Brazil; ^2^ Cardiovascular and Renal Research, Institute of Molecular Medicine, University of Southern Denmark, Odense, Denmark

**Keywords:** Muridae, placentation, Sigmodontinae, trophoblast invasion, uterine spiral artery

## Abstract

Giant cells are a prominent feature of placentation in cricetid rodents. Once thought to be maternal in origin, they are now known to be trophoblast giant cells (TGCs). The large size of cricetid TGCs and their nuclei reflects a high degree of polyploidy. While some TGCs are found at fixed locations, others migrate throughout the placenta and deep into the uterus where they sometimes survive *postpartum*. Herein, we review the distribution of TGCs in the placenta of cricetids, including our own data from the New World subfamily Sigmodontinae, and attempt a comparison between the TGCs of cricetid and murid rodents. In both families, parietal TGCs are found in the parietal yolk sac and as a layer between the junctional zone and decidua. In cricetids alone, large numbers of TGCs, likely from the same lineage, accumulate at the edge of the placental disk. Common to murids and cricetids is a haemotrichorial placental barrier where the maternal-facing layer consists of cytotrophoblasts characterized as sinusoidal TGCs. The maternal channels of the labyrinth are supplied by trophoblast-lined canals. Whereas in the mouse these are lined largely by canal TGCs, in cricetids canal TGCs are interspersed with syncytiotrophoblast. Transformation of the uterine spiral arteries occurs in both murids and cricetids and spiral artery TGCs line segments of the arteries that have lost their endothelium and smooth muscle. Since polyploidization of TGCs can amplify selective genomic regions required for specific functions, we argue that the TGCs of cricetids deserve further study and suggest avenues for future research.

## 1 Introduction

Cricetidae is the second largest family of rodents (Steppan and Schenk, 2017). This diverse group includes the Palaearctic hamsters and Holarctic voles and lemmings but has its greatest radiation in the New World where it occupies many of the niches associated with murid rodents elsewhere (Table 1). Studies in the mouse have determined the origin and diversification of giant cells and identified four distinct phenotypes (Simmons et al., 2007; Hu and Cross, 2010). Cricetid rodents have giant cells that can differ from those of mice in size, mobility, and location. They have attracted the attention of scientists since the 19th and early 20th centuries. Some early workers considered them maternal in origin (see below). However, since the careful work of Orsini in the golden hamster (*Mesocricetus auratus*), it is widely accepted that the giant cells of the cricetid placenta and placental bed are trophoblastic in origin (Orsini, 1954). Giant cells are polyploid due to endoduplication, a process that has been particularly well investigated in the East European grey vole [*Microtus mystacinus* (*M. rossiaemeridionalis*)] (Zybina et al., 2005; Zybina et al., 2009; Zybina et al., 2014).

**TABLE 1:** Subfamilies of cricetid rodents. Number of genera and currently recognized species according to Kelt and Patton (Kelt & Patton, 2020).

Subfamily	Common names	Distribution	Genera	Species
Cricetinae	Hamsters	Palaearctic	7	18
Arvicolinae	Voles, lemmings, muskrat	Holarctic	29	162
Neotominae	New World rats and mice	Largely North American	16	140
Tylomyinae	Vesper rats, climbing rats	Central and South American	4	10
Sigmodontinae	New World rats and mice	Largely South American	86	434

In contrast to voles and hamsters, rather little is known about giant cells in New World cricetids, although some information is available for the South American Sigmodontinae (Favaron et al., 2011; Favaron et al., 2013). Our aim here is to review the evolution of thought about cricetid giant cells and summarize the current state of knowledge. We include some hitherto unpublished findings in sigmodonts and identify potential avenues for further research.

### 1.1 Overview of the cricetid placenta

**FIGURE 1:**
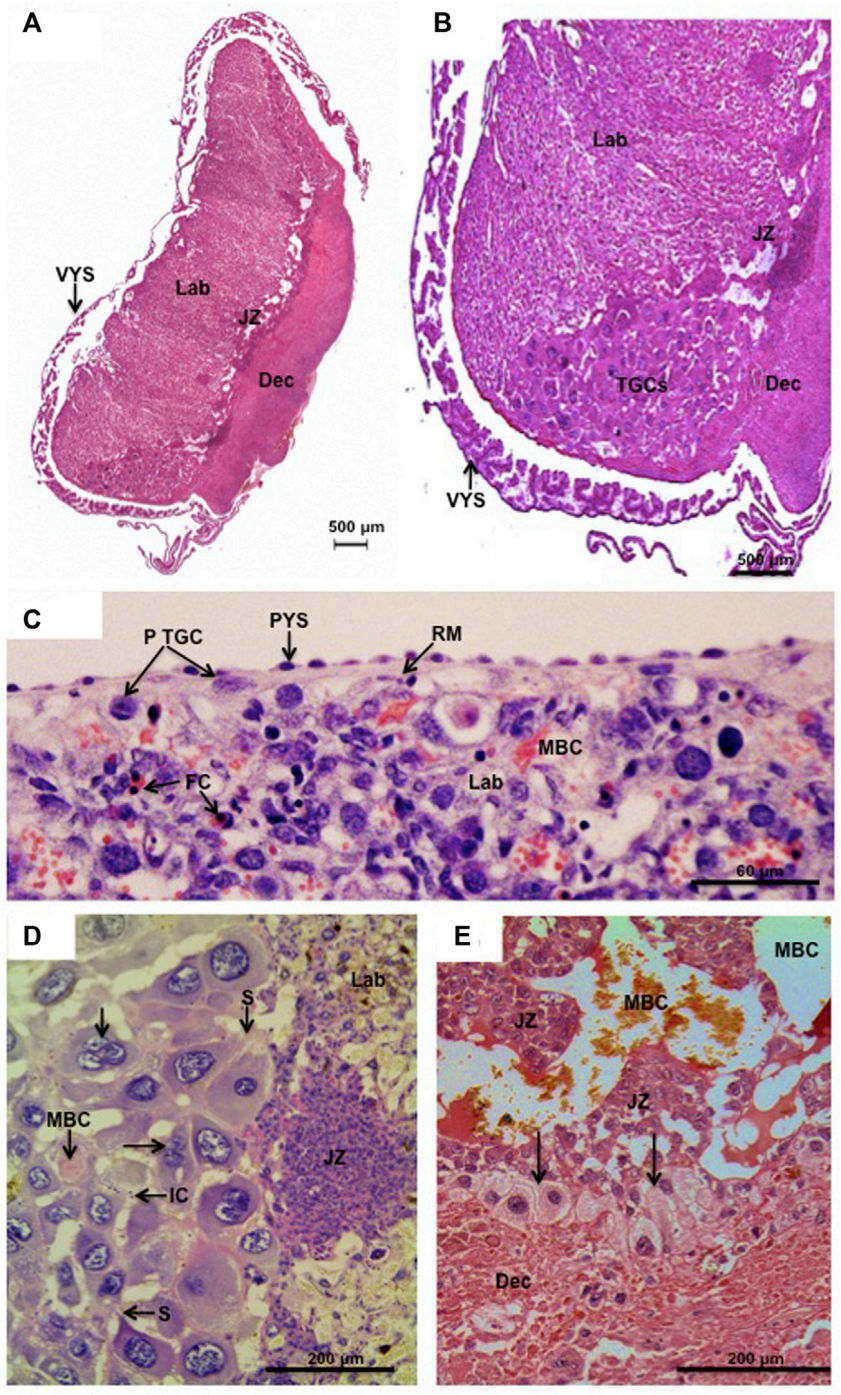
Parietal trophoblast giant cells (p-TGCs). **(A,B)** *Hylaeamys megacephalus.* Histology of the chorioallantoic placenta with the main compartments: labyrinth (Lab), junctional zone (JZ), and decidua (Dec). Note in **(B)** the trophoblast giant cells (TGCs) at the border of the junctional zone (JZ) and decidua (Dec). Visceral yolk sac (VYS) is close to the placental disk. Haematoxylin and eosin. **(C)**. *Necromys lasiurus*. p-TGCs near the surface of the placenta beneath Reichert’s membrane (RM) and parietal yolk sac endoderm (PYS). Lab = labyrinth, MBC = maternal blood channel, FC = fetal capillaries. Haematoxylin and eosin. **(D)** *Hylaeamys megacephalus.* Highly developed p-TGCs are closely associated with maternal blood channels (MBC). Note some bi- and tri-nucleated cells (arrows) and the sprouts (S) that maintain contact between the parietal TGCs. IC = inflammatory cells, JZ = junctional zone, and Lab = labyrinth. Periodic acid-Schiff. **(E)** *Cerradomys subflavus*. p-TGCs (arrows) at the border of the junctional zone (JZ) and underlying the decidua (Dec). MBC = maternal blood spaces. Picrosirius.

The gross anatomy of the placenta is similar in murid and cricetid rodents. It is discoid in shape and the placental barrier is haemochorial. There are three main compartments (Figures 1A, B). The labyrinth is the area of gas and nutrient exchange between maternal blood channels and fetal capillaries. The junctional zone is composed of trophoblast and has maternal blood channels although no fetal vessels. The decidua is derived from the uterine endometrium but also includes invasive trophoblast. In both murid and cricetid rodents, yolk sac inversion is complete. A thin layer of parietal yolk sac covers the outside of the labyrinth (Figure 1C), whilst the visceral yolk sac (Figures 1A, B) attaches near the centre of the disk.

## 2 Historical perspectives

The first thorough study of giant cells in a cricetid rodent was made in the common vole (*Microtus arvalis*) by Joseph Disse (1852–1912), then Professor of Anatomy in Marburg, (Disse, 1906). Disse noted the huge size of the cells (up to 0.24 mm diameter), their distribution throughout the implantation site and their phagocytic properties. He showed them engulfing pieces of the maternal symplasma as well as maternal erythrocytes. Their principal role was to enlarge the implantation chamber, thus making way for growth of the embryo. Disse considered the giant cells to be entirely maternal in origin and derived from the decidua. Importantly, he found giant cells at early stages where the blastocyst still was free in the uterus. Some years later a similar observation was made in the bank vole (*Clethrionomys glareolus*) where two giant cells up to 0.4 mm in diameter were found in the endometrium of a uterus with implanting blastocysts (Brambell and Rowlands, 1936). Sansom also claimed a maternal origin for giant cells in the European water vole (*Arvicola amphibius*), arguing they were derived from hypertrophied endothelial cells (Sansom, 1922). Mossman, however, firmly rejected a maternal origin of cricetid giant cells based on his studies of the musk rat (*Ondatra zibethicus*) (Mossman, 1937). Importantly, he showed the occurrence of two generations of giant cells originating from a current and a previous pregnancy (Figure 31 in (Mossman, 1937)), thus countering the claims of previous authors (Disse, 1906; Brambell and Rowlands, 1936).

So the giant cells of rodents are fetal in origin and accordingly will be referred to as trophoblast giant cells (TGCs). The primary source of TGCs is the mural trophectoderm of the blastocyst. These cells end up as the parietal TGCs found in association with the parietal endoderm and Reichert’s membrane covering the surface of the chorioallantoic placenta in cricetid (Figure 1C) and murid rodents (Hu and Cross, 2010) as well as in other rodents such as the guinea pig (*Cavia porcellus*) (Carter et al., 1998). However, there are just 60 or so cells in the mural ectoderm of the mouse and most of the parietal TGCs as well as other types of giant cell arise from the polar trophectoderm; this was originally characterized as a secondary source (Simmons et al., 2007).

The distinction between primary and secondary giant cells was established for a cricetid, the golden hamster, by Margaret Ward Orsini (1916–2004) (Ward, 1948; Orsini, 1954). The primary giant cells were derived from the trophectoderm at the abembryonic pole of the blastocyst and were responsible for the initial penetration of the uterine epithelium. As this proceeded, the trophectoderm at the embryonic pole developed into the ectoplacental cone or Träger. Vacuolated cells within the ectoplacental cone then enlarged to form the secondary giant cells. At this stage the primary giant cells had begun to migrate into the decidua. The secondary giant cells were not as large but were actively phagocytic (Ward, 1948). Orsini recognized a third source of giant cells likely originating from the trophospongium and migrating to and lining maternal blood vessels (she called them tertiary giant cells) (Orsini, 1954).

The biology of TGCs is better understood in the light of findings in the mouse where gene expression patterns more clearly define cell lineages (see below) (Simmons et al., 2007; Hu and Cross, 2010). We have adopted the resultant notation and feel this to be reasonable since Muridae and Cricetidae are sister groups. Additionally in the mouse, ectoplacental cone explants and trophoblast stem cells differentiate into secondary trophoblast giant cells characterized by their large size and expression of placental lactogen 2 (Pl2). The transcriptome of these cultured TGCs has been examined in some detail (El-Hashash and Kimber, 2004; Hayakawa et al., 2018). This has yet to be attempted in a cricetid rodent.

### 2.1 TGCs are polyploid

It was apparent to early workers that TGCs do not undergo mitosis (Orsini, 1954) and extensive studies in the East European grey vole (*Microtus mystacinus*) have confirmed that TGCs are polyploid, that is, they have undergone multiple rounds of genome duplication without mitosis (Zybina et al., 2014). The molecular and genetic mechanisms involved in the polyploidization of TGCs have been extensively studied in recent years, especially in murine models. In this regard, it is known that polyploidization of TGCs occurs by non-typical cell cycles such as endoreplication and results in an increase of DNA content accompanied by the enlargement of nuclei and expansion of cell size (Zybina et al., 2011). Figure 1D show these morphological characteristics in the parietal TGCs (p-TGCs) of *Hylaeamys megacephalus*. Five amplified regions (two clusters of prolactins, serpins, cathepsins, and the natural killer (NK)/C-type lectin complex) were identified in the mouse p-TGCs. The selective amplification of a number of these genes during endoreplication of the p-TGC genome provides the necessary gene copies for general TGC functions and metabolism that are required for correct placental development (Hannibal and Baker, 2016). In order to understand the mechanisms involved in control, division, and differentiation of TGCs, Buss et al. (2022) showed that although TGCs *in situ* have amplified centrioles (PLK4-dependent), centriole number does not increase exponentially with DNA reduplication. However, the centrioles that do exist are disengaged and separated and become functional centrosomes (four or more), which may be involved in the acquisition of an invasive phenotype in polyploid TGCs that is critical to their ability to migrate during placentation. In addition, the centrioles are part of the microtubule-organizing centre and are important for cell polarity, division, and signalling (Loncarek and Bettencourt-Dias, 2018). It has been shown in the mouse that TGCs are polytenic, a state requiring a deep global histone reorganization, especially of the H2AZ, H2AX and H3.3 variants, during TGC differentiation, which is associated with the formation of a unique chromatin structure in TGCs (Hayakawa et al., 2018). Moreover, cell cycle regulators, such as p57kip2 and cyclin E1/E2, are involved in TGC polyploidization. Thus, it was demonstrated in knockout mice that cyclins E1 and E2 are essential for endoreplication of TGCs. Although TGCs were able to differentiate into giant cells *in vitro* (showing that cyclins E1 and E2 are dispensable for cell proliferation), they lost the ability to undergo multiple rounds of DNA synthesis, showing that the lack of endoreplication was a cell-autonomous defect (Parisi et al., 2003). In the mouse, differentiation of precursor cells occurs first in the ectoplacental cone and later in the spongiotrophoblast layer. This process is regulated by bHLH transcription factors encoded by the genes *Hand 1*, which is commonly related to TGC differentiation (Riley et al., 1998), but seems to be more involved in the regulation of TGC maturation (Chakraborty and Ain, 2018), and *Mash 2*, which is required for the maintenance of giant cell precursors, since its overexpression prevents differentiation (Scott et al., 2000). Furthermore, TGC formation by differentiation of trophoblast stem cells and the development of polyploidy in murids (rat and mouse) are controlled by precocious over-expression of *Nostrin* (Nitric Oxide Synthase Trafficking Inducer) which leads to up-regulation of members of the prolactin (*Prl*) gene family that are markers associated with invasion (*Prl4a1*, *Prl2a1*) and TGC development (*Prl2c2*, *Prl3d1*, *Prl3b1*) as well as increasing the number of polyploid TGCs that arise by endoreduplication (Chakraborty and Ain, 2018). In summary, the development of TGCs during gestation is rigorously orchestrated by different genes and signalling molecules, resulting in different degrees of polyploidy among TGCs, which affect both functional and physiological characteristics of the different lineages.

## 3 Giant cell distribution

The primary giant cells are derived from mural trophectoderm. Secondary giant cells occur in diverse locations. For the mouse, clarity has emerged from studies of gene expression that can identify cell lineages. Whilst such data are lacking for cricetids, we employ a notation derived from those studies.

### 3.1 Classification in murid rodents

Current opinion on the origins of TGC lineages in the laboratory mouse is based on gene expression studies, as it has been demonstrated that TGC subtypes have distinct patterns of gene expression, including some specific markers (Simmons et al., 2007). In addition to the secondary parietal TGCs, the polar trophectoderm of the mouse gives rise to *Tpbpa* ^−^ cells that are the source of several lineages (*Tpbpa* codes for trophoblast-specific protein alpha). One of these is *Tpbpa* ^+^ and is the source of glycogen trophoblast cells, some of the parietal TGCs, and spiral artery associated TGCs. Thus, *Tpbpa* has been considered a key marker gene of the precursors of invasive TGCs (Woods et al., 2018) and ablation of *Tpbpa* ^+^ cells in the mouse placenta results in insufficient trophoblast invasion and committed maternal spiral artery remodeling (Hu and Cross, 2011). A separate lineage, which remains *Tpbpa* ^−^, gives rise to the three layers of trophoblast that line maternal blood channels in the labyrinth. The outermost layer is comprised of cytotrophoblast, and these cells have been designated sinusoidal TGCs. Finally, the giant cells which line the maternal blood channels that supply the labyrinth, called canal TGCs, may be derived from *Tpbpa* ^−^ or *Tpbpa* ^+^ cells or both (Simmons et al., 2007; Hu and Cross, 2010). To varying degrees murid TGCs express genes for placental lactogens 1 and 2 (*Pl1* and *Pl2*), proliferin (*Prf*) and a placenta-specific cathepsin (*Ctsq*) (Simmons et al., 2007). Gene expression by TGCs has yet to be studied in a cricetid rodent. The following analysis therefore considers the categories defined for the mouse in the light of morphological and histochemical evidence from cricetids.

### 3.2 Primary TGCs

These TGCs are derived from the mural trophectoderm (Ward, 1948). They are phagocytic and have been ascribed a role in enlargement of the implantation chamber (Disse, 1906) although they soon migrate into the decidua (Ward, 1948). Whilst differentiation of such cells occurs early in the mouse (Müntener and Hsu, 1977) they have not been characterized as phagocytic at the earliest stages although they become so later (Fawcett et al., 1947). Migration of TGCs from this locus has been noticed in the mouse as early as E7.5 (Reinius, 1967) much as in the hamster (Ward, 1948).

### 3.3 Parietal TGCs

Parietal TGCs are found near the surface of the placenta beneath Reichert’s membrane (Figure 1C). They are also lined up at the border of the junctional zone and abut the underlying decidua (Figures 1B, E). Murid and cricetid placentas are similar in these respects and in the mouse the parietal TGCs express *Pl1*, *Pl2* and *Plf*. An additional feature of cricetid placentas, that we have not seen described for murids, is an accumulation of TGCs at the edge of the labyrinth and junctional zones. This was first observed by Sansom in the water vole (Sansom, 1922) and is also a prominent feature in Sigmodontinae such as rice rats (*Cerradomys*, *Euryoryzomys* and *Hylaeamys*) (Favaron et al., 2011) (Figures 1B, D). In this area, parietal TGCs reach their highest degree of development and polyploidy, including bi- and tri-nucleated cells, as observed in the placenta of Azara’s rice rat (*Hylaeamys megacephalus*) (Figure 1D). Projections or sprouts ensure a close contact between the parietal TGCs in the placental margin of cricetid species, and small maternal blood spaces with some inflammatory cells can be seen among them (Figure 1D). In the rat, the sprouts produced by TGCs are cytokeratin-positive in nature and allow these cells to phagocytose decidual cells and to sustain the continuous layer at the border with decidua (Zybina et al., 2011).

### 3.4 Sinusoidal TGCs

**FIGURE 2:**
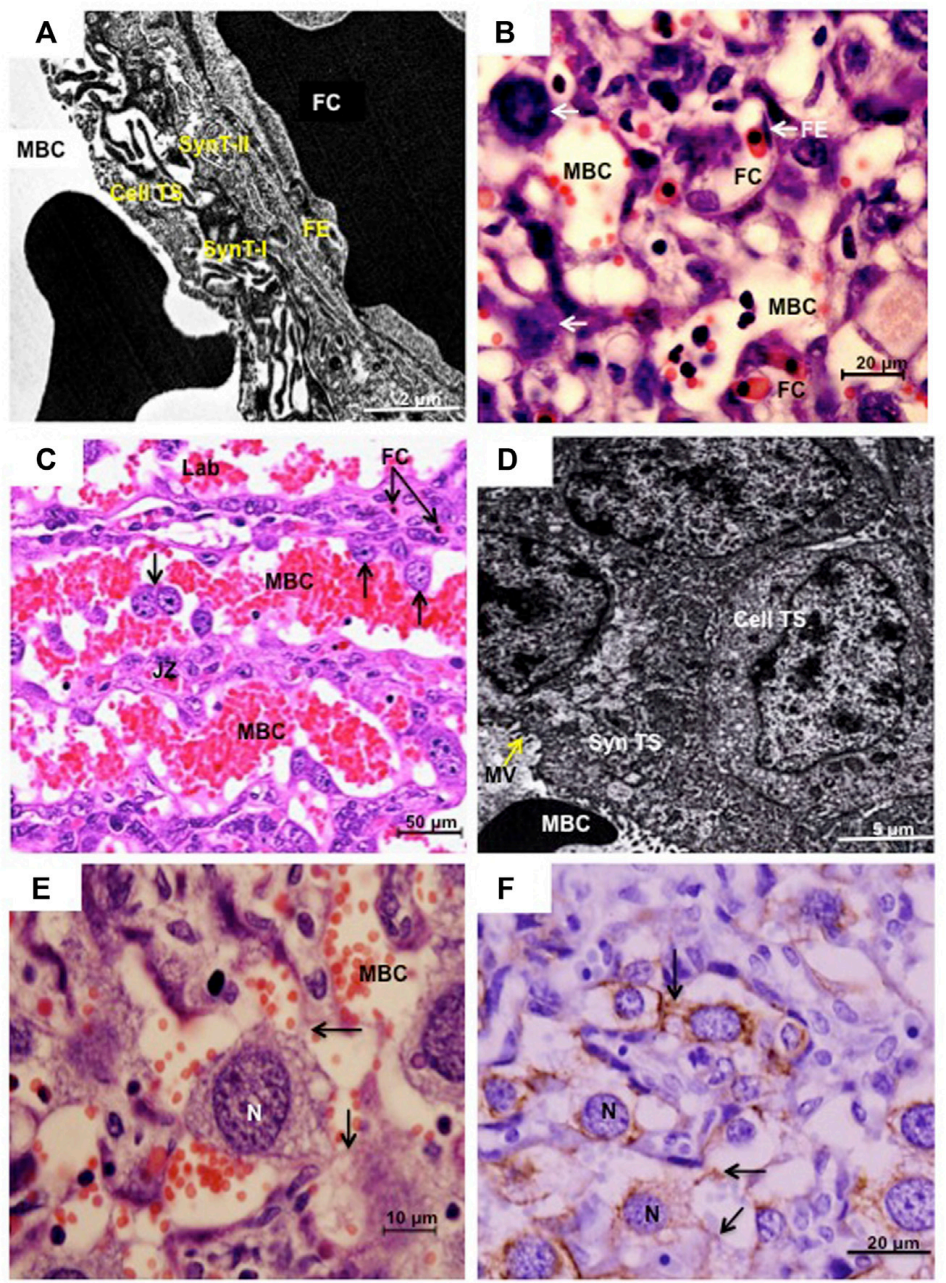
**(A)** and **(B)** Sinusoidal trophoblast giant cells in *Necromys lasiurus*. Transmission electron microscopy (TEM) and histology (haematoxylin and eosin), respectively. The placental barrier is composed of two inner syncytial layers (SynT-I and SynT-II) and an outer cytotrophoblast layer (Cell TS) facing the maternal blood channel (MBC). FE = fetal endothelium, FC = fetal capillary; arrows = sinusoidal trophoblast giant cells. **(C)** and **(D)** Canal trophoblast giant cells in *Cerradomy subflavus* and *N. lasiurus*, respectively. **(C)** Maternal blood channels (MBC) in the junctional zone (JZ) are lined by TGCs (arrows). Fetal capillaries (FC) are restricted to the labyrinth compartment (Lab). Haematoxylin and eosin. In these areas, syncytial trophoblast (Syn TS) with microvilli (MV) in the surface lined the MBC, associated with underlying cellular trophoblast (Cell TS). TEM. **(E)** and **(F)**. *N. lasiurus*. Canal TGCs with large rounded central nuclei (N) and cytokeratin-positive cytoplasmic projections (arrows). Haematoxylin and eosin and cytokeratin, respectively.

The placenta of cricetid rodents is haemotrichorial as has been shown in a variety of species by transmission electron microscopy (Figure 2A) (Enders, 1965; Carpenter, 1972; King and Hastings, 1977; Favaron et al., 2011; Martinez et al., 2020). The layer facing the maternal blood sinuses is made up of cytotrophoblasts that only recently were recognized to be giant cells. Beneath them are two layers of syncytiotrophoblast (Figure 2A). Of note, there is considerable variation in the interhaemal barrier of rodents and haemotrichorial placentation is a derived state known only from murid and cricetid rodents (Mess and Carter, 2009). According to Woods et al. (2018), sinusoidal TGCs in the mouse form a fenestrated and discontinuous layer that does not constitute a complete barrier. The sinusoidal TGCs also formed a discontinuous layer in the species we examined, so that the two layers of syncytiotrophoblast constitute the barrier through which nutrients and gases must be transported to reach the fetal blood circulation and the embryo (Woods et al., 2018). The interhaemal barrier has been described at the ultrastructural level in four subfamilies of cricetid rodent. In addition to our work, species examined are the golden hamster (Cricetinae) (Carpenter, 1972, 1975); the Eastern deer mouse (*Peromyscus maniculatus*) (Neotominae) (King and Hastings, 1977); Norway lemming (*Lemmus lemmus*), collared lemming (*Dicrostonyx groenlandicus*), red-backed vole (*Clethrionomys rutilus*) and Taiga vole (*Microtus xanthognathus*) (Arvicolinae) (King and Hastings, 1977); and hispid cotton rat (*Sigmodon hispidus*) (Martinez et al., 2020). The overall picture is that gaps or lacunae occur in the outermost layer of trophoblast.

Redefinition of the cytotrophoblasts as sinusoidal TGCs was based in part on an observed increase in nuclear size and DNA content with advancing gestation in the mouse (Coan et al., 2005) and in part on expression of *Pl2* and *Ctsq* (Simmons et al., 2007). The DNA content was lower than in parietal TGCs (Coan et al., 2005) and the cytotrophoblasts seemed to adopt some of the characteristics of giant cells rather than being derived from a classical TGC lineage. In addition to the known volume increase related to the polyploidization, it was demonstrated that the volume fraction of sinusoidal TGCs increased from early (6.1%) to late gestation (19.3%) in the hairy-tailed akodont (*Necromys lasiurus*) (Favaron et al., 2013). The picture is further complicated in cricetids by the occurrence of some much larger TGCs within the labyrinth.

### 3.5 Canal TGCs

The uterine spiral arteries continue as maternal blood canals that cross the junctional zone and labyrinth before branching to supply the maternal sinusoids. These canals are lined by trophoblast. In the mouse, the lining consists largely of TGCs, known as maternal blood canal-associated TGCs (Hu and Cross, 2011), which express *Pl2* and *Plf* (Simmons et al., 2007) In sigmodonts, we found these channels to be lined largely by syncytiotrophoblast (Favaron et al., 2011) although there also were prominent TGCs (Figure 2D). In *Necromys lasiurus*, for example, these cells have a large rounded central nucleus (Figure 2E) with cytoplasmic projections that stain for cytokeratin and keep the canal TGCs connected to each other (Figure 2F).

### 3.6 Spiral artery associated TGCs

**FIGURE 3:**
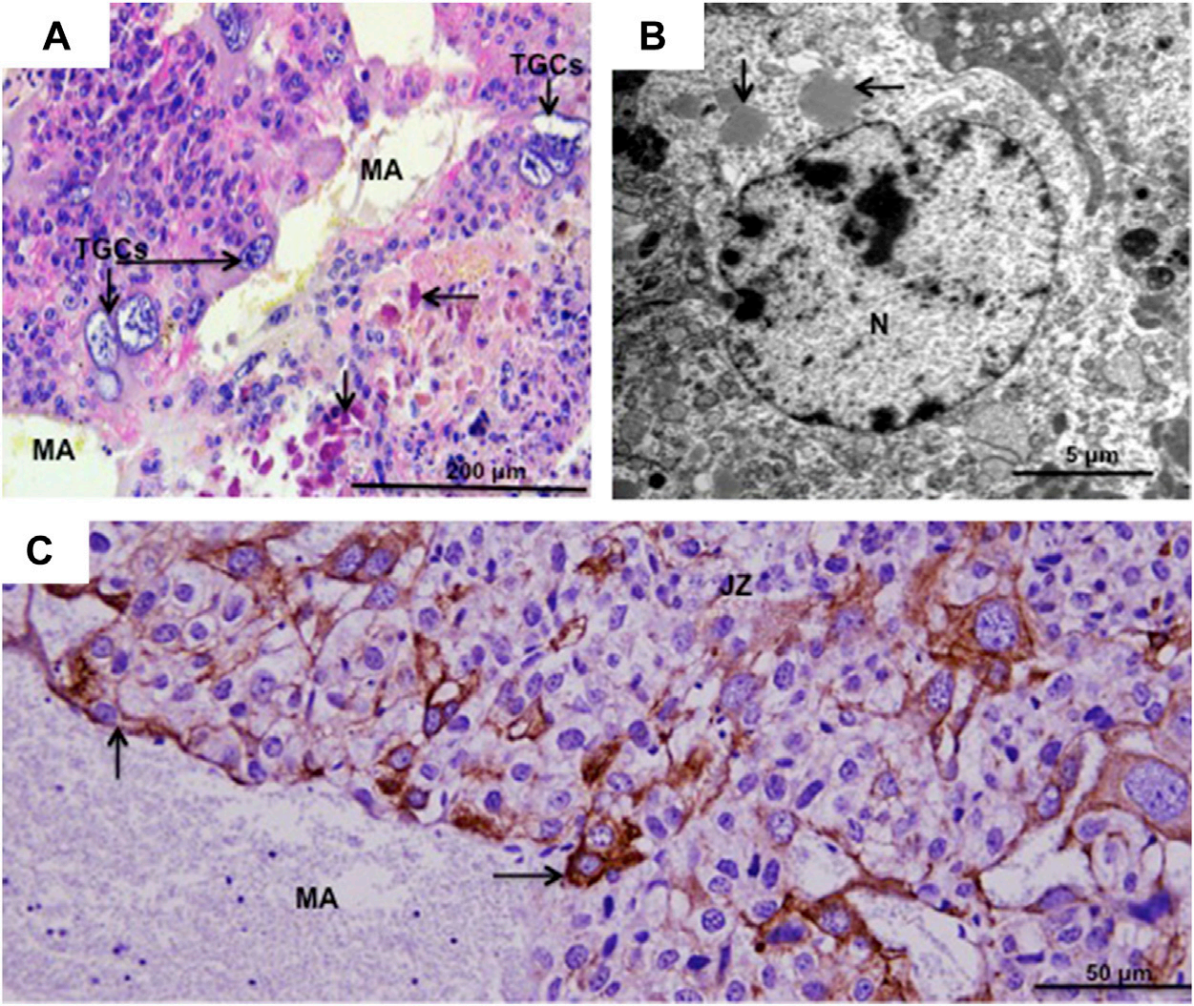
Spiral artery trophoblast giant cells. **(A)** *Hylaeamys megacephalus.* Maternal artery (MA) surrounded by uterine natural killer cells and large trophoblast giant cells (TGCs). Arrows = PAS-positive granules in uterine natural killer cells. Periodic acid-Schiff. **(B)**. *Euryoryzomys* sp. Granules (arrows) in the cytoplasm of uterine natural killer cells. N = nucleus. Transmission electron microscopy. **(C)** *Necromys lasiurus*. Maternal artery (MA) near the junctional zone (JZ) lined by large TGCs (arrows) and without the smooth muscle layer and endothelium. Cytokeratin.

In the hamster, invasion of the uterine arteries by TGCs started on day 7 of pregnancy (term was 16 days). Orsini referred to them as sheathed arteries (Orsini, 1954). It is now apparent that the sheaths consist in large part of uterine natural killer (uNK) cells, which are vimentin positive and contain granules that stain for the periodic acid-Schiff reaction, as shown for *Hylaeamys megacephalus* (Figure 3A), and by transmission electron microscopy for *Euryoryzomys* sp. (Figure 3B). They also stain for the glycan bound by *Dolichos biflorus* (DBA) lectin (fucosylated α-N-acetyl glucosamine) as previously demonstrated for *Cerradomys subflavus* (Favaron et al., 2011).

In the hamster, Pijnenborg and colleagues distinguished two waves of TGC invasion by the endovascular route. The first occurred between 8 and 12 days and proceeded *via* arteries circumferential to the implantation site reaching as far as the mesometrial arteries. The second wave from day 12 onward proceeded *via* the central spiral artery (Pijnenborg et al., 1974). While the latter authors ascribed a major role to TGCs in the transformation of the spiral arteries, more recent work in mice and rats suggests that the uNK cells are at least as important in this respect (Croy et al., 1996).

In *Hylaeamys megacephalus*, maternal arteries in the decidua are surrounded by uNK cells (PAS-positive) (Figure 3A). The continuations of these vessels, that supply the canals of the junctional zone and labyrinth, exhibit extensive remodelling; they lack the smooth muscle layer and endothelium and are instead lined by large TGCs as observed in *H. megacephalus* (Figure 3A) and *Necromys lasiurus* (Figure 3C).

### 3.7 Migratory TGCs of cricetid rodents

There are numerous observations in cricetid rodents of TGCs migrating to placental and uterine tissues that do not readily fit into the categories defined for the mouse. They have been seen in the water vole (Sansom, 1922), bank vole (Ozdzenski and Mystkowska, 1976), golden hamster (Parkening, 1976), short-tailed field vole (*Microtus agrestis*) (Copp and Clarke, 1988), Chinese or striped dwarf hamster (*Cricetulus barabensis*) (Blankenship et al., 1990), and large vesper mouse (*Calomys callosus*) (Ferro and Bevilacqua, 1994).

**FIGURE 4:**
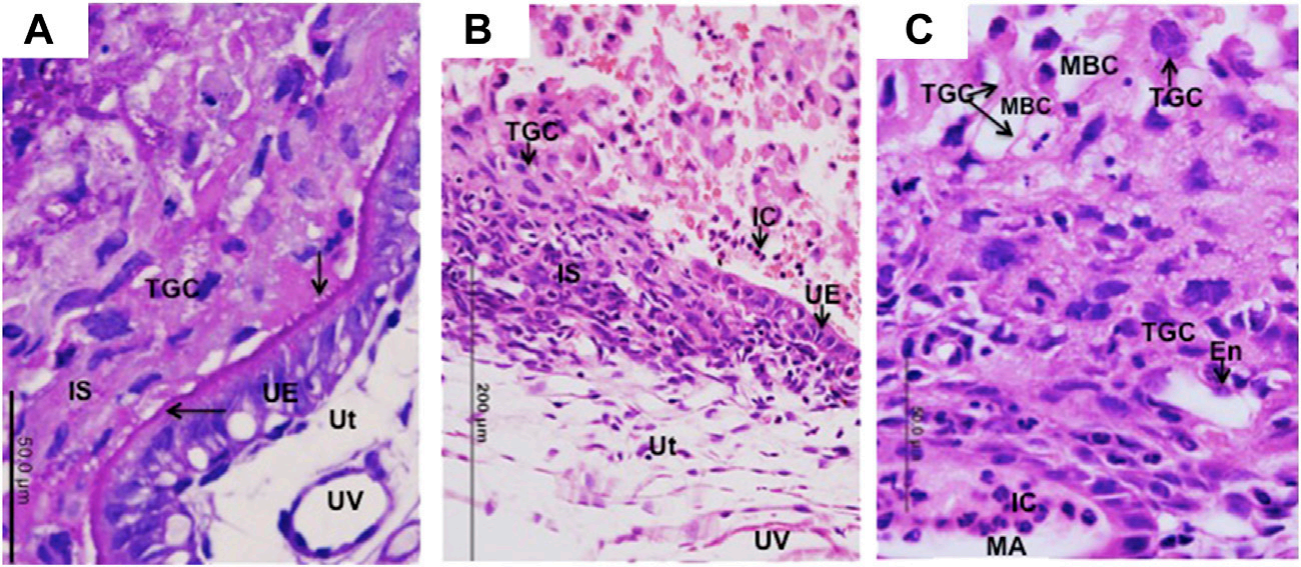
Migration of TGCs in *Necromys lasiurus*. **(A–C)** Early contact (day 10) between trophoblast giant cells (TGC) and uterine tissues (Ut) at the implantation site (IS). **(A)** PAS-positive reaction in the apical surface of intact uterine epithelium (UE). Note the initial establishment of contact (arrows) between trophoblast giant cells (TGCs) and uterine epithelium (UE). UV = uterine vessels. Periodic acid-Schiff. **(B)** A region still with some intact uterine epithelium (UE) with invasive TGCS (to the side or below). IC = inflammatory cells. Haematoxylin and eosin. **(C)** Trophoblast giant cells (TGCs) lining the maternal blood channels (MBC). Note the migration of trophoblast giant cells (TGCs) into maternal tissues associated with maternal vessel endothelium (En) and inflammatory cells (IC) within maternal artery (MA). Haematoxylin and eosin.

In golden hamster, the first contact between maternal and fetal cells occurs by late day 3 (Parkening, 1976), in the vesper mouse on day 4 (Ferro and Bevilacqua, 1994), and in the dwarf hamster on day 6 (Blankenship et al., 1990). Although the establishment of interactions between maternal and fetal tissues was not chronologically followed, we observed different degrees of migration of TGCs into maternal tissues at implantation sites of *Necromys lasiurus* from days 10–11. At this stage of development, in some areas TGCs are in contact with intact maternal tissues (PAS-positive endometrial epithelium) (Figure 4A). In other areas, TGCs lead the invasion process through the uterine epithelium (Figure 4B) going deep towards the basal lamina to reach the maternal circulation in deeper areas (Figure 4C). In these areas of invasiveness, TGCs promote the phagocytosis of maternal tissues, and disrupt maternal blood vessels. TGCs can be observed associated with the maternal arteries, which frequently contain inflammatory cells (Figure 4C). Unlike murids, the interaction between maternal and fetal cells proceeds rapidly; and there is no interdigitation between the trophoblast microvilli and uterine epithelium in cricetids (Ferro and Bevilacqua, 1994).

## 4 Conclusion

The functions of TGCs are often difficult to divine even when transcriptomic data are available (Hu and Cross 2010). Parietal TGCs are thought to have an endocrine function since they express *Plf* and *Pl2* in the mouse. Placental lactogens play an important role in maintenance of pregnancy (Soares, 2004) and proliferin promotes angiogenesis in the placental labyrinth (Jackson et al., 1994). We speculate that the large accumulation of p-TGCs at the placental margin in cricetids is related to their endocrine functions.

### 4.1 Comparison of cricetid and murid rodents

The location of TGCs in cricetid rodents is shown in Table 2. In comparison to mouse, two features deserve comment. Parietal TGCs occur in expected locations such as below Reichert’s membrane in the parietal yolk sac and as a layer broadly delineating the border between junctional zone and decidua. In cricetids, however, TGCs of that layer are expanded at the placental margin to an area several cells thick. This was first illustrated for the water vole (Arvicolinae) (Figure 27 in Sansom, 1922). It occurs also in the golden hamster (Cricetinae) (Pijnenborg, 1975) and we have described it in several species of Sigmodontinae (Favaron et al., 2011). To our knowledge no equivalent structure is known for murid rodents.

**TABLE 2:** Provisional notation for trophoblast giant cells (TGCs) of cricetid rodents with comparison to the mouse for which transcriptional data is also available.

Notation	Location	Comparison to mouse	Further comments
Primary TGCs	Derived from mural trophectoderm and migrate to decidua	Similar	Phagocytic and may enlarge implantation chamber (Disse, (1906)
Parietal TGCs	Parietal yolk sac beneath Reichert’s membrane; distinct layer between junctional zone and decidua; accumulation at placental margin	Identical location in parietal yolk sac and between junctional zone and decidua	Accumulation at margin described for several families of cricetids (Sansom, (1922); Pijnenborg, (1975); Favaron et al. (2011), but not in mouse or rat
Sinusoidal TGCs	Maternal-facing layer of the interhaemal barrier in the labyrinthine zone	Identical location	Best characterized in the mouse (Coan et al. (2005); may have acquired the characteristics of TGCs rather than being derived from a TGC lineage
Canal TGCs	Part of the lining of the canals in the junctional zone and labyrinth	Similar location but constitute most of the lining	Difference between cricetids and mouse may be questioned (see text) (Hu and Cross, (2011); Favaron et al. (2011)
Spiral artery associated TGCs	Large TGCs lining the transformed spiral arteries	Similar location	Proposed role in spiral artery transformation (Pijnenborg et al. (1974), although uNK cells are more important (Croy et al. (1996)
Migratory TGCs	Found in numerous locations including the uterine wall where they can survive *postpartum*	Apart from early migration towards the spiral arteries, murine TGCs seem to wander less than in cricetids	Prominent feature of cricetid placentation (Sansom, (1922); Ozdzenski and Mystkowka, (1976); Parkening, (1976); Copp and Clarke, (1988); Blankenship et al. (1990); Ferro and Bevilacqua, (1994)

In the mouse, canal TGCs were said to line the maternal blood channels that supply the sinusoids of the exchange area (Simmons et al., 2007). In sigmodonts, we found these canals to be lined by syncytiotrophoblast interspersed with TGCs. Close study of the relevant micrographs from the mouse study (Simmons et al., 2007) revealed that only some nuclei expressed *Plf* and *Pl2*. Therefore, we sought for further information on the lining of canals in murine placenta. As others have remarked, there are surprisingly few papers on the ultrastructure of mouse placenta (Coan et al., 2005). In contrast, a thorough study of the rat placenta has shown that maternal channels in the junctional zone are lined by two types of trophoblasts. Firstly, by small basophilic cells (spongiotrophoblasts) and secondly, by giant cells (Davies and Glasser, 1968).

### 4.2 Future perspectives

Placentation varies greatly between different mammalian taxa (Mossman, 1937; Carter and Enders, 2016). Fifteen per cent of living mammals are cricetid rodents (Mammal Diversity Database, 2022). Thus, we argue that they deserve further study. Moreover, although structural aspects of the placenta are rather similar between cricetids and murids, quantitative dynamics, including TGC quantification (Favaron et al., 2013), suggest important differences that perhaps are related to optimization of maternal-fetal exchange or other reproductive adaptations.

A clear priority for future work on cricetid TGCs must be to study gene expression analogous with what has been done in the mouse. Recently the genomes of several cricetids have been sequenced including, from subfamily Arvicolinae, the North American water vole (*Microtus richardsoni*), montane vole (*M*. *montanus*) (Duckett et al., 2021), and narrow-headed vole complex (*Lasiopodomys* spp.) (Petrova et al., 2022); from subfamily Cricetinae, the Siberian hamster (*Phodopus sungorus*) (Bao et al., 2019); and from subfamily Neotominae, the white-footed mouse (*Peromyscus leucopus*) (Long et al., 2019). To our knowledge genomic data is lacking for Sigmodontinae although it is the most speciose subfamily. A recent essay on transcriptomics in the endocrine organs of the Siberian hamster (*Phodopus sungorus*) did not include the placenta but does indicate what may be possible (Stewart et al., 2022). In addition, transcriptome data was recently used to understand the molecular mechanisms of intraspecies differentiation in narrow-headed voles, the focus being on reproductive isolation. A list of genes involved in processes of reproduction (for example *Cited1*, *Nostrin* and *Stk3*), which play a role in regulation of cell differentiation during placental development, was revealed by the EggNOG-mapper (Petrova et al., 2022). Hannibal and Baker (2016) using whole-genome sequencing and digital droplet PCR of mouse p-TGCs, showed that polyploidy can affect gene regulation by amplifying a subset of genomic regions required for specific functions, such as: haematopoiesis and vascular remodelling (prolactins); embryo development and survival and placental abnormalities (serpins); placental invasion by degradation of extracellular matrix proteins (cathepsins); and pregnancy success through trophoblast invasion and vascular remodelling (uNK/C-type lectin complex). The authors also suggested that TGC subtypes could have different regions of amplification corresponding to specialized cellular functions, which makes the cricetids an interesting model due to their specific TGC populations.

A deeper knowledge of the biology of TGCs in cricetid rodents will contribute not only to clarify evolutionary aspects of maternal-fetal communication, but may increase our knowledge in different fields: 1) maternal tolerance and immunomodulation during trophoblast invasion and vascular remodelling; 2) how changes of gene copy number and their expression affect normal and abnormal cell differentiation, for which TGCs are an interesting model; 3) the distribution of histone variants such as H3.3, in the genome of TGCs; 4) the development of knockout models for genes of interest (including exclusive markers for specific TGC lineages), which could generate data regarding placental abnormalities; 5) since the placental trophoblasts are responsible for fetal protection, and infection of these cells is directly involved in the pathogenesis of several intracellular parasites, knowledge of TGC diversity in cricetids will be helpful for the establishment of effective laboratory models, as has been shown in *Calomys callosus* (Costa et al., 2009; Rosa et al., 2022); and finally 6) exploration of specific TGC-lineages as models for cancer development and metastasis, especially using cell cultures and 3D models.
